# Computational Study of Abdominal Aortic Aneurysm Walls Accounting for Patient-Specific Non-Uniform Intraluminal Thrombus Thickness and Distinct Material Models: A Pre- and Post-Rupture Case

**DOI:** 10.3390/bioengineering11020144

**Published:** 2024-01-31

**Authors:** Platon Sarantides, Anastasios Raptis, Dimitrios Mathioulakis, Konstantinos Moulakakis, John Kakisis, Christos Manopoulos

**Affiliations:** 1 Laboratory of Biofluid Mechanics & Biomedical Technology, School of Mechanical Engineering, National Technical University of Athens, 157 72 Zografos, Greece; ppsarantides@mail.ntua.gr (P.S.); raptistasos@mail.ntua.gr (A.R.); 2 School of Engineering, Bahrain Polytechnic, Isa Town P.O. Box 33349, Bahrain; dimitrios.mathioulakis@polytechnic.bh (D.M.); 3 Department of Vascular Surgery, School of Medicine, University of Patras, 265 04 Patras, Greece; moulakakis@upatras.gr (K.M.); 4 Department of Vascular Surgery, Attikon University Hospital, National and Kapodistrian University of Athens, 106 79 Athens, Greece; kakisis@med.uoa.gr (J.K.)

**Keywords:** abdominal aortic aneurysm, intraluminal thrombus, patient-specific geometry, local Cartesian coordinate system, finite element analysis, Holzapfel–Gasser–Ogden model, Mooney–Rivlin model

## Abstract

An intraluminal thrombus (ILT) is present in the majority of abdominal aortic aneurysms, playing a crucial role in their growth and rupture. Although most computational studies do not include the ILT, in the present study, this is taken into account, laying out the whole simulation procedure, namely, from computed tomography scans to medical image segmentation, geometry reconstruction, mesh generation, biomaterial modeling, finite element analysis, and post-processing, all carried out in open software. By processing the tomography scans of a patient’s aneurysm before and after rupture, digital twins are reconstructed assuming a uniform aortic wall thickness. The ILT and the aortic wall are assigned different biomaterial models; namely, the first is modeled as an isotropic linear elastic material, and the second is modeled as the Mooney–Rivlin hyperelastic material as well as the transversely isotropic hyperelastic Holzapfel–Gasser–Ogden nonlinear material. The implementation of the latter requires the designation of local Cartesian coordinate systems in the aortic wall, suitably oriented in space, for the proper orientation of the collagen fibers. The composite aneurysm geometries (ILT and aortic wall structures) are loaded with normal and hypertensive static intraluminal pressure. Based on the calculated stress and strain distributions, ILT seems to be protecting the aneurysm from a structural point of view, as the highest stresses appear in the thrombus-free areas of the aneurysmal wall.

## 1. Introduction

An abdominal aortic aneurysm (AAA) is a pathology of the aorta, irreversible and mostly asymptomatic, in which the physiological diameter increases by at least 50%. It affects 7–9% of the male population over 65 years old [1] and is considered a high risk disease, as the mortality rate reaches 90% in the case of rupture. An AAA is treated by surgical intervention to restore the blood flow and drastically reduce the possibility of rupture. There are two available interventional methods: (1) open aneurysm repair (OAR) and (2) endovascular aneurysm repair (EVAR), with the latter being considered more effective due to the reduced 30-day mortality rate compared to the former [2]. Both interventional methods may be accompanied by various complications that increase the risk during implementation [3,4]. Therefore, the treating surgeon must assess the patient’s condition and compare the risk of surgery to the risk of rupture. The decision-making parameter to assess the risk of rupture is the maximum transverse diameter, with an AAA requiring surgical intervention when its maximum diameter reaches 5.5 cm for males and 5.0 cm for females [5,6,7]. In recent years, efforts have been made to upgrade the decision-making process and enrich it with several histological, biological, and mechanical parameters. Factors such as active smoking [8], gender [9,10], peak wall stress (PWS) [11,12], and wall shear stress (WSS) [13,14,15] have been examined, among others, and evaluated.

The intraluminal thrombus (ILT), both its presence and extent, plays a crucial role in the evolution of the AAA. Constituted by a 3D fiber matrix containing red blood cells, platelets, and blood proteins, the ILT attaches to the aorta’s luminal layer, diminishing its natural healing ability by obstructing endothelial cell coverage and replacing the fibrin network with collagen [16]. The ILT is considered to induce inflammation via hypoxia, weakening the aortic wall, enabling larger deformations [17], and affecting the distribution of stresses [18]. The ILT is not always included in analyses found in the open literature, despite being present in 75% of aneurysms [19]. Moreover, when the ILT is considered in the relevant calculations [20,21,22], the conclusions are often conflicting regarding its impact on the stress distribution. Some studies suggest that the ILT functions as a means of a mechanical shield for the wall (“cushioning effect”) [20,23], while others dispute that claim [24]. The incorporation of the ILT into AAA analyses has been the focus of many previous studies. In the analysis of Di Martino et al. [25], an asymmetric geometry of the thrombus was created using Gaussian distribution curves. Hyperelastic, homogeneous, isotropic material models were assigned both to the wall and to the thrombus, and a uniform intraluminal pressure was applied. A series of static simulations were conducted assuming different values for the ILT mechanical parameters. The case of a bilayered ILT, consisting of the luminal and external layers, was also examined. Similarly, Toungara et al. [26] created idealized axisymmetric AAA geometries defined by a “parabolic-exponential shape function” with a constant wall thickness. For a given wall geometry, several models were created that vary in terms of ILT accumulation within the AAA, and in each of them, four cases were created combining two wall-modeling approaches (hyperelastic isotropic and anisotropic) with two thrombus-modeling approaches (dense hyperelastic and porohyperelastic). In all cases, transient flow and pressure simulations were conducted. In an analogous way, Polzer et al. [27] generated three axisymmetric AAAs with different maximum diameters and a constant wall thickness. After modeling the wall as a homogeneous hyperelastic material and the ILT as poroelastic, an in vivo-mimicking pressure wave was applied, providing insights into the influence of a poroelastic ILT on the computed wall stress.

Alternatively, other studies account for the real (non-idealized) geometry of the wall. Arslan et al. [28] constructed three distinct artificial thrombus volumes of uniform thickness as an intrusion of the luminal layer of the wall, significantly altering the intraluminal volume. In each case, the ILT was modeled as a linear elastic material, while the aneurysmal wall was modeled as a Mooney–Rivlin material. In all cases, fluid–structure interaction (FSI) simulations were performed, and the impact of the thrombus on biomechanical factors, such as PWS and WSS, was examined. Similarly, Molony et al. [29] examined the stresses and hemodynamics of three geometries, a healthy aorta, an AAA, and an operated AAA (EVAR intervention), using FSI. A normal aorta was reconstructed from computed tomography (CT) scans, and a strain–energy function (from the work of Raghavan et al. [30]) was fitted based on the data of seven patients. Likewise, the diseased and operated AAA geometries were reconstructed from CT scans: the first consisted only of the aneurysmal wall, while the latter included both the wall and ILT geometries. An isotropic hyperelastic model (proposed by Raghavan [31]) was applied to both diseased wall geometries, while for the ILT, a two-parameter, hyperelastic, isotropic material model was applied. In all cases, velocity and pressure waveforms were used as boundary conditions at the inlet and outlet, respectively.

Finally, other researchers have employed fully patient-specific AAA geometries, reconstructing both the wall and the thrombus from CT scans. Doyle et al. [32] constructed a patient-specific 3D AAA model by segmenting each entity via a brightness threshold method using a commercial DICOM file processing software. To the same 3D model (with constant wall thickness), several material properties were applied, such as linearly elastic and hyperelastic models for the aortic wall and likewise for the ILT, while static intraluminal pressure was applied. Following a very similar method, Rissland et al. [33] reconstructed a patient-specific AAA geometry and conducted an FSI simulation assuming a linearly elastic ILT and anisotropic hyperelastic wall models according to [34]. Similar geometry reconstructions were performed by Di Martino et al. [35], as well as by Auer et al. [36], utilizing research-based software for boundary contour recognition. In the comprehensive work of Doyle et al. [37], the AAA wall and ILT geometries were reconstructed from CT scans, while tissue samples from both entities were extracted for further examination. Both sample types were subjected to uniaxial mechanical testing, whereas thickness measurements of the wall tissue were used to create a varying-thickness digital-twin wall geometry. Uniaxial testing was used for the fitting of two-parameter and three-parameter isotropic hyperelastic material models to the wall and the ILT, respectively. A structural Finite Element Analysis (FEA) was carried out with a uniform intraluminal pressure of 120 mmHg, based on which Von Mises wall stress and principal strain distributions were predicted. It is important to state that from the aforementioned studies, only [33,36,37] reconstructed AAA geometries with partial accumulation of the ILT. Also worth mentioning is the work of de Lucio et al. [38], in which the implementation of the Holzapfel–Gasser–Ogden (HGO) material model was performed on idealized aneurysmal wall geometries consisting of three distinct layers, representing the intima, media, and adventitia of the aorta. To each of the three layers, different material parameters were assigned, and the aneurysmal geometries were loaded with a uniform intraluminal pressure of 120 mmHg, ending up with predictions of the stress distributions and displacement fields.

In the current study, the influence of non-uniform ILT accumulation on the mechanical behavior of the AAA was assessed via a method/workflow developed using open software tools and libraries. Two AAA geometric models, before and after rupture, originating from a single patient were reconstructed from medical imaging data, distinguishing the ILT from the aortic wall based on suitable software and a procedure that is described in detail. Moreover, each of the latter components was assigned distinct material properties for the structural analysis based on FEA. Namely, the ILT was modeled as an isotropic linear elastic material, and the arterial wall was modeled as (a) the Mooney–Rivlin hyperelastic model (for the unruptured geometry) and (b) the HGO model (for both geometries), in which the proper orientation of the collagen fibers was implemented by introducing suitable local Cartesian coordinate systems. Moreover, suitable contact conditions were applied at the interface of the two entities (ILT and aorta wall).

## 2. Materials and Methods

The implementation of the method relied primarily on the use of open software. Throughout all stages of the method, namely, the processing of medical images, reconstruction, the processing and smoothing of geometries, meshing, numerical solving, and post-processing, open software and tools were utilized. These tools allowed for the customization and adaptation of the method to suit the specific needs of the problem. Moreover, in the results-processing stage, they allow for the calculation and presentation of any desired quantities. The workflow begins with the processing of the CT scans and the creation of the AAA geometric models using 3D Slicer (v5.6.0) [39] and Meshmixer (v.3.5) (Autodesk Inc., San Francisco, CA, USA), followed by the generation of the mesh and the establishment of the local coordinate systems on the aortic wall in MATLAB R2023a, academic license (MathWorks Inc., Natick, MA, USA) utilizing the GIBBON toolbox (v3.5.0) [40]. The FEA was carried out in FEBio (v3.8) software [41] through GIBBON.

### 2.1. Clinical Case

The examined clinical case is a patient with a ruptured AAA, substantiated by CT scans carried out both before and after the rupture, spaced 1 year and 8 months apart. The anonymized records are courtesy of the Department of Vascular Surgery at the University Hospital of Patras and have secured the requisite approval from the Ethics Committee for the secondary use of the medical imaging data. At 85 years, the patient’s initial assessment indicated an unruptured AAA, measuring a peak transverse diameter of 75 mm. By age 87, upon hospital admission, this diameter had expanded to 88 mm, coinciding with an AAA rupture.

**Figure 1:**
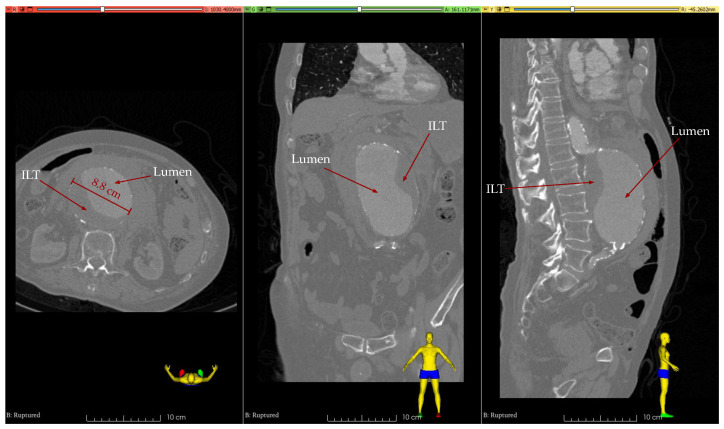
CT images after AAA rupture: axial, coronal, and sagittal views (from left to right).

The AAA is categorized as infrarenal with a fusiform shape. Within the AAA, there is a noticeable accumulation of an ILT, which is distributed non-uniformly. Specifically, as seen in Figure 1, its presence is minimal on the anterior side, in contrast to the posterior and left sides, where it reaches up to 28 mm in thickness. Calcifications are also prominent in several areas of the aortic wall but were not taken into account in the structural analysis.

### 2.2. Segmentation, Reconstruction, and Smoothing

The CT scans were imported into 3D Slicer in DICOM format to segment the regions of interest and reconstruct the corresponding surfaces. The accurate representation of the ILT geometry is a key part of the modeling. Calcifications were treated as components of the aortic wall rather than distinct entities. The AAA region was specifically isolated to the infrarenal section of the abdominal aorta, extending roughly 4 cm below the aortic bifurcation along the iliac arteries.

The methodology described below is rooted in the concept of boundary surfaces. In this approach, the individual entities (aortic wall and ILT) are delineated by their respective 3D (boundary) surfaces that encapsulate them. This particular approach diverges from the reconstruction of the thrombus and the wall as distinct 3D bodies. The reason for adopting this approach is that, after smoothing, the ILT and the aortic wall will no longer share a common interface, thus hindering the definition of a contact condition between the two entities.

**Figure 2:**
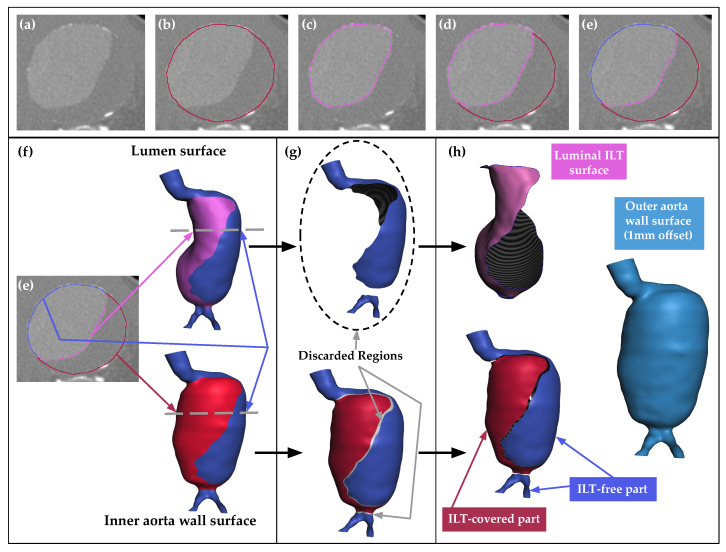
Indicative annotated cross-sections of (**a**) the sagittal view of the aneurysm in the absence of masking, (**b**) the whole AAA’s inner surface (blood lumen and ILT included) in red, (**c**) the blood lumen mask in pink, (**d**) both masks, (**e**) both masks viewed with the masking intersection highlighted in blue. (**f**) The reconstructed lumen and inner aortic wall surfaces, (**g**) the discarded regions, including the part of the lumen surface not in contact with the ILT (**top**) and strip regions along the border between the ILT-covered and the ILT-free parts of the inner aortic wall surface (**bottom**), (**h**) the final surfaces to be stitched together, including the luminal surface of the ILT (the part of the lumen surface in contact with the ILT), the fragmented inner aortic wall surface (composed of the ILT-covered and the ILT-free parts), and the outer aortic wall surface (created by offsetting the inner aortic wall surface by 1 mm before discarding the strip regions).

Figure 2a depicts an indicative cross-section of the AAA. Utilizing 3D Slicer’s tools, two masks were created on this section: one referring to the inner surface of the aortic wall, including both the lumen and the ILT (Figure 2b), and the other referring only to the lumen (Figure 2c). As is evident in Figure 2d, an ILT-free region exists, at which the two masks intersect. The ILT surface in contact with the blood is defined as the luminal ILT surface (depicted in Figure 2d with pink color), and that in contact with the aortic wall is named the external ILT surface (depicted in Figure 2d with red color). Since the ILT is identified as the volume between its luminal surface and its external surface, the volume of the ILT is equal to zero at the intersecting region of the above two masks (blue area in Figure 2e). This area will cause issues in the meshing stage, since it is not possible to create zero-volume tetrahedral elements. However, this problem was solved by employing the concept of fragmenting the boundary surfaces into individual pieces, which are then assembled in a way that allows both meshing and the correct representation of the individual geometries.

The 3D boundary surfaces of the lumen and the inner aortic wall, once reconstructed, are not smooth enough. To rectify this, after importing them into the Meshmixer environment in STL format, a smoothing step was undertaken using the available tools, resulting in two smooth surfaces: the lumen and the inner aortic wall (Figure 2f). Several approaches to the reconstruction of the aortic wall can be found in the literature. Some studies have reproduced a wall of varying thickness based on medical imaging [42,43], while others have adopted a constant thickness [35,44]. Here, since the aortic wall could not be clearly identified within the CT images, it was modeled with a constant thickness of 1 mm, as determined by necropsy studies on AAAs containing an ILT [45]. The outer surface of the aortic wall was generated by offsetting its inner surface by a fixed value of 1 mm, representing the thickness of the wall (visible with light-blue color in Figure 2h).

In Meshmixer, a thickness analysis was performed to detect and eliminate areas where the two surfaces coincide. With regard to the lumen surface, its area, which was identified in the thickness analysis as being in contact with the inner aortic wall, was removed, resulting in the luminal ILT surface (Figure 2g top). As for the inner aortic wall, its common boundaries with the luminal ILT surface were selected and erased, thus creating a discontinuity that outlines the boundaries of the thrombus (see relevant strips in Figure 2g bottom). Three surfaces appear in the present application, namely, the lumen, the inner aortic wall, and the outer aortic wall surfaces (Figure 2h). In Figure 2h, the fragmented surface of the inner aortic wall is depicted, consisting of two areas: one marked in red, representing the ILT-covered region, and the other one marked in blue, representing the ILT-free regions.

### 2.3. Meshing

**Figure 3:**
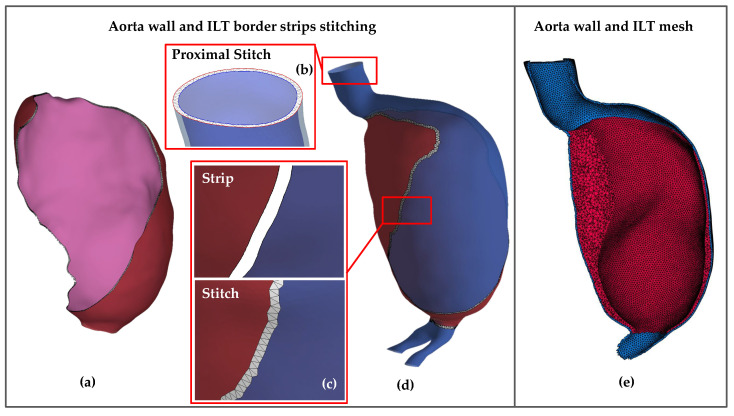
(**a**) Stitch between the luminal (pink) and external (red) surfaces of the ILT, (**b**) stitch (planar triangulated surface) along the proximal end edges, between the inner (blue) and outer (light blue) surfaces of the aortic wall, (**c**) detail of fragmented surfaces before (**top**) and after (**bottom**) performing a stitch (white triangles), between the ILT-covered and the ILT-free parts of the inner surface of the aortic wall, (**d**) stitched inner surface of aortic wall, (**e**) tetrahedral mesh of aortic wall (blue) and ILT (red).

As a result of the previous processes, the aorta and ILT surfaces are fragmented and disconnected. To generate a spatial mesh for FEA, both the ILT and the aortic wall need to be closed surfaces that share a common interface. In this respect, the following procedure was followed using GIBBON toolbox: the surfaces, imported in STL format, were remeshed with uniform triangles, eliminating areas with variable triangle sizes, which result from Meshmixer’s smoothing and brushing tools. To connect two surfaces, the nodes belonging to their edges were identified and joined using the Delaunay triangulation method (Figure 3c). This process introduces new surface triangles, creating a unified surface, and henceforth is referred to as “stitching”, with each individual connection termed a “stitch”.

For the internal and external ILT surfaces, one stitch was performed at the luminal boundaries and another was performed at its distal end, right before the aortic bifurcation (Figure 3a). With respect to the inner aortic wall, stitches were also performed at the two strips between its ILT-covered and the ILT-free parts, as well as at three other areas, namely, at the proximal end (infrarenal aorta, Figure 3b) and two distal ends (two iliac arteries) of the aortic wall, Figure 3d.

Once these strips are sealed, two distinct volumes emerge—one for the ILT and another for the aortic wall. A surface-smoothing process follows, with the aim of assimilating the stitches and making them smooth and uniform. The two closed surfaces of the thrombus and the aortic wall share a region with common facets and nodes that constitute the actual interface between them. This interface is used in later stages to define the contact condition between the two materials. The meshing was performed using the TetGen library, creating two separate tetrahedral meshes with unique identification numbers, which enables the assignment of materials with different properties to each entity (Figure 3e).

### 2.4. Local Material Axes

For the modeling of the aortic wall, the HGO model was adopted, which describes a complex hyperelastic material composed of a ground matrix and collagen fibers [46]. The orientation of the fibers within the wall plays a crucial role in its mechanical behavior. For this reason, defining the fiber orientation on the surface of the aneurysm by a vector field is necessary. Since the longitudinal axis of the AAA is not rectilinear, changing its orientation in space, the definition of local coordinate Cartesian systems (LCCSs) on the vessel wall is necessary. An LCCS is parametrically defined as follows.

**Figure 4:**
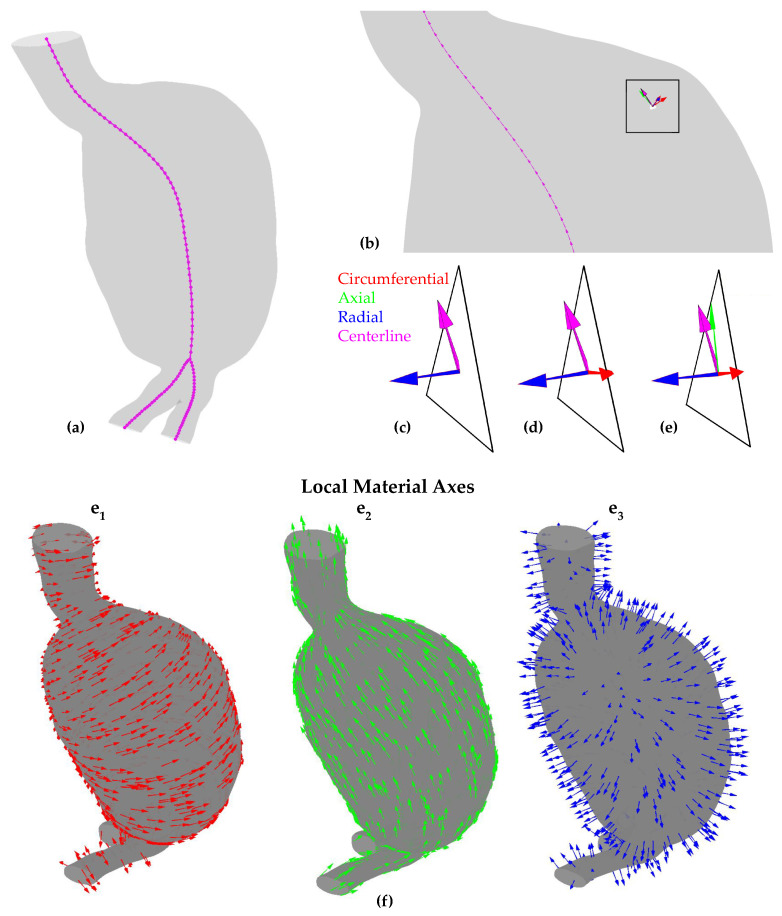
(**a**) Centerline points, (**b**) centerline vector (${\vec{a}}_{centr}$), (**c**) centerline vector (${\vec{a}}_{centr}$) and radial component (${\vec{e}}_{3}$), (**d**) circumferential component (${\vec{e}}_{1}$) tangent to the triangle, (**e**) axial component (${\vec{e}}_{2}$) tangent to the triangle, (**f**) components of the local coordinate systems.

Firstly, the concept of the AAA centerline was employed, which was extracted using 3D Slicer’s VascularToolkit algorithms (Figure 4a). The centerline extraction algorithm uses a Voronoi diagram to compute the medial axis and then traces the branches from user-defined endpoints. After suitable resampling, a set of 90 points was obtained, with 30 points below the aortic bifurcation and 60 above. A centerline vector (${\vec{a}}_{centr}$) is defined as the vector connecting two consecutive points on the centerline, with a direction from the iliacs toward the inlet of the aneurysm (Figure 4b).

Secondly, the centroids of the triangular sides of the tetrahedral mesh elements constituting the aortic wall’s external surface (referred to as “external triangles” of the “external tetrahedra”) were calculated. These centroids were then matched with the nearest points on the centerline, creating pairs of centroids and centerline points for the entire external surface of the aorta wall.

To define the LCCS, three mutually orthogonal unit vectors are calculated: circumferential (${\vec{e}}_{1}$), axial (${\vec{e}}_{2}$), and radial (${\vec{e}}_{3}$). The last one is the normal unit vector on each external triangle (Figure 4c).

The circumferential component (${\vec{e}}_{1}$) is calculated as the cross product of the centerline vector (${\vec{a}}_{centr}$) and the radial component (${\vec{e}}_{3}$) (Figure 4d). Namely,

(1) $${\vec{e}}_{1}={\vec{a}}_{centr}\times {\vec{e}}_{3}$$

Finally, the axial component (${\vec{e}}_{2}$) is computed as the cross product of the radial and circumferential components (Figure 4e):

(2) $${\vec{e}}_{2}={\vec{e}}_{3}\times {\vec{e}}_{1}$$

The origin of each LCCS is positioned at the center of mass of the corresponding external tetrahedra. The remaining mesh elements of the wall adopt the LCCS of the nearest external tetrahedron. The result of the above process is a set of LCCSs, with two axes being tangent to the aortic wall (axial and circumferential) and the third being perpendicular to it, as visualized in Figure 4f.

### 2.5. Material Model

The AAA is modeled in the present study as a composite of two distinct structures: the aortic wall and the ILT. Each structure is modeled with different material models.

#### 2.5.1. Intraluminal Thrombus

For the ILT, the energy equation of deformation for a hyperelastic material is assigned, which, for small deformations, reduces to a classical linear elastic material:

(3) $$W=\frac{1}{2}\lambda {\left(trE\right)}^{2}+\mu E:E$$

where **E** is the Euler–Lagrange strain tensor, and λ and μ are the Lamé parameters related to the elastic modulus (*E*) and the Poisson’s ratio (ν) through the equations:

(4) $$\lambda =\frac{E\nu }{\left(1+\nu \right)\left(1-2\nu \right)}$$

(5) $$\mu =\frac{E}{2\left(1+\nu \right)}$$

The values of *E* and ν (see next paragraph) were adopted from the experimental study of Di Martino et al. [47], who examined thrombotic AAAs.

#### 2.5.2. Aortic Wall

##### HGO Model

For the aortic wall, the decoupled strain–energy function Ψ is

(6) $$\Psi =\Psi_{g}\left(C\right)+\sum_{i=4,6}\Psi_{fi}\left(C,H_{i}\right)$$

The strain–energy function $\Psi_{g}$ represents the ground matrix

(7) $$\Psi_{g}\left(C\right)=\frac{c}{2}\left(I_{1}-3\right)$$

where *c* is a parameter of the ground matrix and

(8) $$I_{1}=tr\left(C\right)$$

is the first invariant. The strain energy of the fibers $\Psi_{fi}$ is given by the following equation:

(9) $$\Psi_{fi}\left(C,H_{i}\right)=\frac{k_{1}}{2k_{2}}\left[exp(k_{2}{\langle E_{\alpha }\rangle }^{2}-1)\right]$$

where the strain of the fibers is enclosed in Macaulay brackets to indicate that the content takes the value of zero for compressive loads, while for tensile loads, it is defined as

(10) $$E_{\alpha }=\kappa \left(I_{1}-3\right)+\left(1-3\kappa \right)\left(I_{4i}-1\right)$$

where κ is a fiber dispersion parameter. $I_{4i}$ is expressed as

(11) $$I_{4i}=M_{i}\cdot C\cdot M_{i},\;\;i=4,\;6\;$$

and $M_{i}$ represents the two collagen-family orientations that are tangent to the aortic wall and symmetrical to the circumferential component by an angle (α),

(12) $$M_{4}=\cos\alpha \cdot e_{1}+\sin\alpha \cdot e_{2}$$

(13) $$M_{6}=\cos\alpha \cdot e_{1}-\sin\alpha \cdot e_{2}\;\;\;\;$$

##### Mooney–Rivlin Model

Alternatively, a two-parameter hyperelastic Mooney–Rivlin material model is applied to the aneurysmal wall, where the strain–energy function (Ψ) is defined in Equation (14). $I_{1}$ and $I_{2}$ are the first and second invariants of the Cauchy–Green deformation tensor, and $c_{1}$ and $c_{2}$ are the model parameters derived from [28,48].

(14) $$\Psi =c_{1}\left(I_{1}-3\right)+c_{2}\left(I_{2}-3\right)$$

The final material models’ parameters for the ILT and aortic wall are presented in Table 1. From the mechanical tests conducted both by Di Martino et al. [47] (related to the thrombus) and by Niestrawska et al. [49] (related to the aneurysmal wall), certain strength limits have emerged. Specifically, from the 21 samples of the ILT that underwent uniaxial tensile tests in [47], an average ultimate tensile strength (UTS) was determined as follows:

(15) $$\sigma_{UTS}^{ILT}=0.085\;\mathrm{MPa}$$

Similarly, a UTS limit was calculated for the aneurysmal aortic wall. From the experimental biaxial tension curves [49], those related to (two) ruptured AAAs were selected. From these curves, four limits were derived, namely, two for each direction [49]. This process resulted in two average stress limits: one axial and another in the circumferential direction:

(16) $$\sigma_{axial}=\frac{\sigma_{axial\_1}+\sigma_{axial\_2}}{2}=0.0895\;\mathrm{MPa}$$

(17) $$\sigma_{circ}=\frac{\sigma_{circ\_1}+\sigma_{circ\_2}}{2}=0.0593\;\mathrm{MPa}$$

Finally, the overall strength limit is calculated according to the Von Mises equation for plane loading, and the final value is obtained as

(18) $$\sigma_{UTS}^{WALL}=\sqrt{\sigma_{circ}^{2}-\sigma_{circ}\sigma_{axial}+\sigma_{axial}^{2}}=\;0.079\;\mathrm{MPa}$$

### 2.6. Finite Element Analysis

The aortic wall and ILT domains were reconstructed from the two AAA medical image datasets (before and after rupture, as mentioned in Section 2.1) and meshed with 4-node tetrahedral elements. FEA simulations were conducted using the open solver FEBio [41]. In each case, the initial geometry, reconstructed from the image processing of CT scans corresponding to a time mean pressure of 100 mmHg, was assumed to be unloaded and stress-free (following the conventional procedure), which is one of the limitations of this study. Regarding the boundary conditions, the nodes of the distal and proximal ends of the aneurysm were constrained in all three degrees of freedom in order to simulate the tethering of the AAA with the rest of the arterial network and surrounding tissue. FSI was not employed, assuming the vessel walls to be of low compliance due to aging (the disease is common for ages above 60). Between the two bodies (ILT and aortic wall), a contact condition was applied to their interface, enforcing a common displacement of these surfaces. A contact force $f_{cont}=\varepsilon_{p}g$ was applied, where **g** is the gap between the two surfaces, and $\varepsilon_{p}$ is a penalty factor calculated automatically, representing the stiffness of the springs inserted between the two surfaces. A quasi-static structural analysis was performed, and a uniform pressure was applied to the inner surface of each AAA, gradually increasing from 80 to 120 mmHg (physiological case) and 200 mmHg (hypertensive case) in forty equal steps, facilitating the convergence of the numerical solution. Stress tensor (σ), strain tensor (ε), and displacement (**d**) data were saved at each time step. The post-processing of the output files was performed using the GIBBON toolbox. The principal stresses ($\sigma_{1},\sigma_{2},\sigma_{3}$) and equivalent strains ($\varepsilon_{1},\varepsilon_{2},\varepsilon_{3}$) were calculated for every tetrahedron, resulting in stress and strain distributions across the volume of the aneurysm.

## 3. Results

### 3.1. Mesh Independence Study

A sensitivity analysis was performed by varying the number of tetrahedral elements of the unruptured geometry from 247,948 (coarse case) to 427,252 (fine case) and to 559,152 (finer case). As shown in Table 2, compared to the fine case that was finally adopted, in the coarse case, the maximum stress was 2.18% smaller, and in the finer case, it was 1.42% larger.

### 3.2. Finite Element Analysis Results

#### 3.2.1. HGO Model

**Figure 5:**
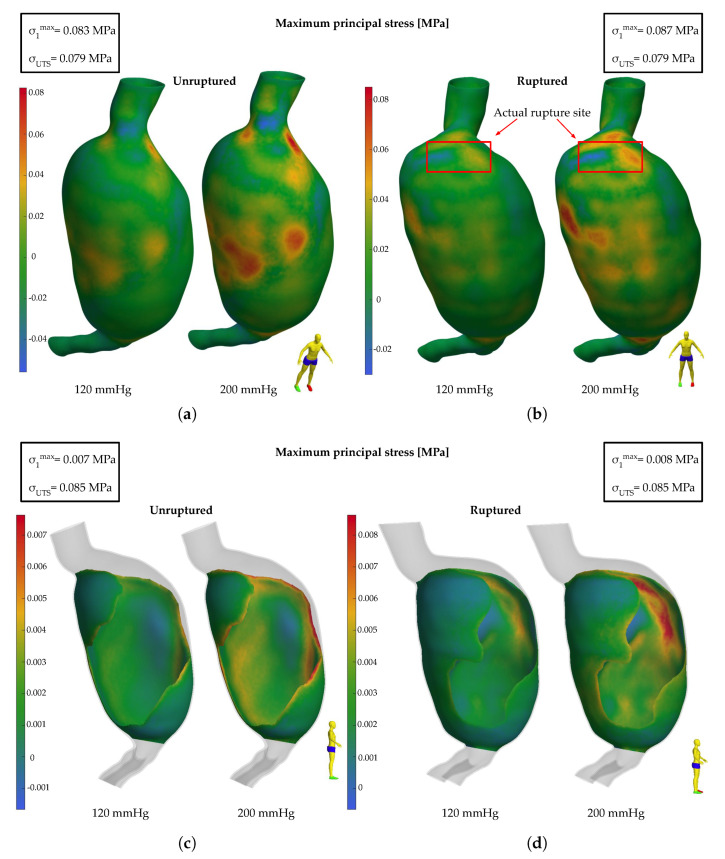
Maximum principal stresses at the aneurysmal wall (**a**) prior to rupture and (**b**) after rupture (in the red box is the region of the rupture) and at the ILT (**c**) prior to rupture and (**d**) after rupture.

Employing the tools provided by GIBBON, maximum principal stress plots ($\sigma_{1}$) were generated for the unruptured and ruptured AAA geometries to depict the mechanical loading of the aortic wall and the thrombus. In Figure 5, the stress patterns are depicted for intraluminal pressures of 120 and 200 mmHg, and their highest values are compared with the previously calculated strength thresholds. Distinct regions with elevated stress concentrations are observed for both the aortic wall and the thrombus. With regard to the unruptured case (Figure 5a), such stress concentrations are predominant on the anterior side of the aneurysm, close to its proximal (aortic neck) and distal ends (aortic bifurcation), and scattered around the maximum AAA diameter area. Concerning the ruptured case (Figure 5b), stress concentrations are likewise pinpointed on its anterior side, most notably in the proximity of the aortic neck region (where the rupture occurred) and at the aortic bifurcation. The zone of the highest stresses is evident in the middle of the aneurysmal bulge, on the right side. The peak stress regions are practically the same between the 120 and 200 mmHg loading scenarios, with only their values being increased with pressure. The thrombus loading is also presented under both loading scenarios (120 and 200 mmHg) for both clinical AAA states (unruptured and ruptured) in Figure 5c,d. In the unruptured case, the highest stresses in the ILT concentrate at the boundaries of the thrombus, where its thickness is minimum, with the values being significantly below the ILT strength limits. A similar pattern emerges in the ruptured case, where the maximum stresses in the ILT are located on the left anterior side of the AAA near the thrombus boundaries.

**Figure 6:**
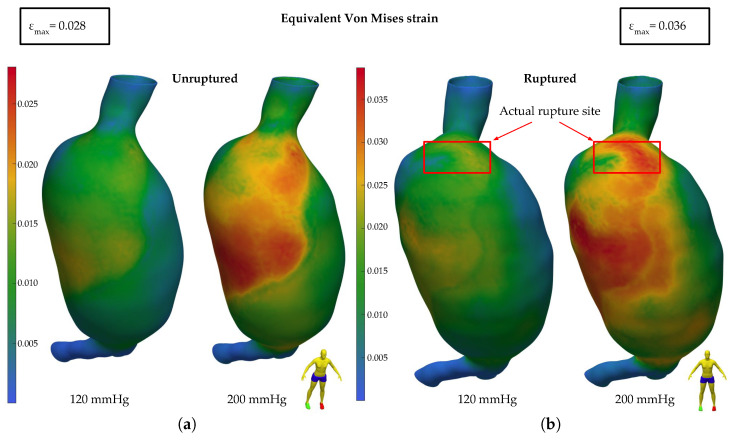
Equivalent Von Mises strain at the aneurysmal wall (**a**) prior to rupture and (**b**) after rupture.

In a similar manner, in Figure 6, the equivalent Von Mises strains are depicted on the outer surface of the aortic wall. In both the unruptured (Figure 6a) and ruptured (Figure 6b) AAA cases, the regions of high strain values clearly align with the ILT-free areas of the aortic wall, localized on the right anterior side of the aneurysmal bulge. The actual rupture site, which was not covered by the ILT, exhibits high strain values, and the maximum value was found around the maximum-aneurysm-diameter region.

**Figure 7:**
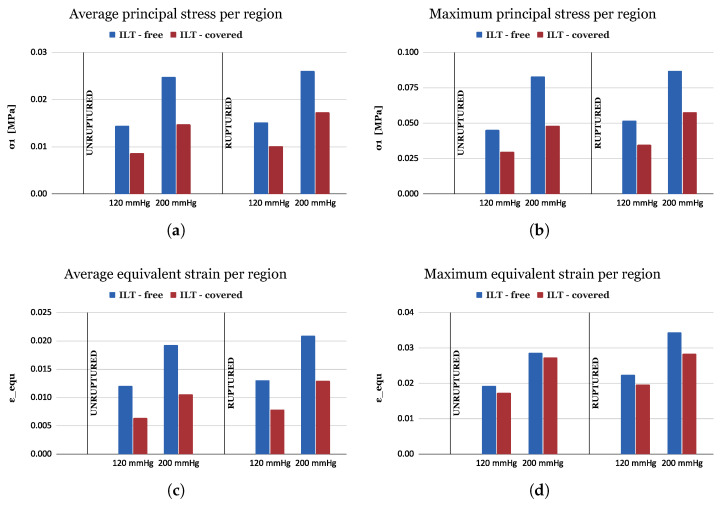
Bar plots of stress and strain values per aortic wall region, ILT-free (blue) and ILT-covered (red), for ruptured and unruptured geometries and pressures of 120 mmHg and 200 mmHg. (**a**) Average principal stress $\sigma_{1}$, (**b**) max principal stress $\sigma_{1}$, (**c**) average equivalent Von Mises strain, and (**d**) max equivalent Von Mises strain.

In Figure 7, the average and maximum values of the principal stress $\sigma_{1}$ (Figure 7a,b) and equivalent Von Mises strain $\varepsilon_{equ}$ (Figure 7c,d) are presented for the ILT- free (blue bars) and ILT-covered (red bars) areas of the aortic wall, for two pressure values (120 mmHg and 200 mmHg), and for two AAA clinical states (unruptured and ruptured). From 120 mmHg to 200 mmHg, both average stresses (Figure 7a) and strains (Figure 7c) are elevated on the order of 70% and 60%, respectively, whereas the clinical state seems to have less influence on these quantities, with a slight increase (about 10% and 15%) from the unruptured to the ruptured case. Comparing the wall areas where there is thrombus with the thrombus-free areas reveals that the first are characterized by significantly smaller stresses (Figure 7a) and strains (Figure 7c) (about 55% and 70%, respectively).

#### 3.2.2. Mooney–Rivlin Model

**Figure 8:**
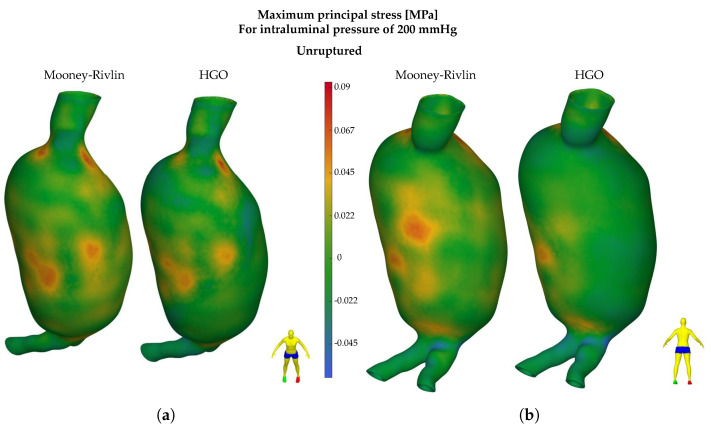
Maximum principal stresses at the aneurysmal wall for intraluminal pressure of 200 mmHg and two different material models (Mooney-Rivlin, HGO) for (**a**) unruptured geometry anterior side and (**b**) unruptured geometry posterior side.

**Figure 9:**
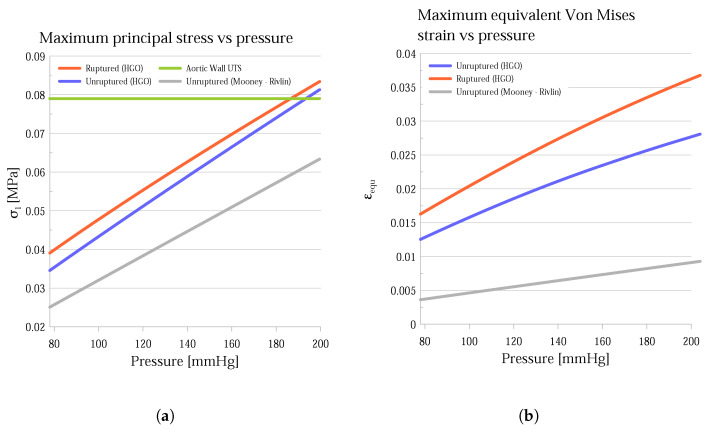
(**a**) Aortic wall maximum principal stress of the ruptured (orange), HGO model of the unruptured (blue), and Mooney–Rivlin model of the unruptured (gray) geometry (aortic wall UTS in green). (**b**) Aortic wall maximum equivalent strain of the ruptured (orange), HGO unruptured (blue), and Mooney–Rivlin unruptured (gray) geometry.

Alongside the HGO model, a widely adopted isotropic hyperelastic material model for the aortic wall, the Mooney–Rivlin model with two parameters, was also examined, as expressed by Equation (14). In contrast to the HGO, this does not involve a composite material, and the contribution of the collagen fibers to the mechanical behavior of the wall is disregarded. This model was applied only to the intact geometry of the wall, while the thrombus, as in the previous case, was expressed as a linear elastic material. Figure 8 presents the distributions of maximum principal stresses in the intact geometry for both the Mooney–Rivlin (left) and HGO (right) models for a pressure of 200 mmHg, and Figure 9 presents the maximum principal stress variation with pressure. Similarly, the maximum Von Mises equivalent strain is also plotted against the applied pressure in Figure 9b, in which the ruptured geometry manifests notably elevated levels. A fundamental observation depicted in Figure 9 is that the highest values of principal stress are distinctly lower in the Mooney–Rivlin model compared to the HGO. However, the mean value of the principal stress for the Mooney–Rivlin is higher compared to that for the HGO. As a result, in Figure 8, concerning the Mooney–Rivlin results, stress concentrations do not reach high values, but they cover larger areas, whereas, in the case of the HGO, higher stress values are recorded on the surface of the wall, but only locally, with the stress values decreasing noticeably in the neighboring areas. In addition, as illustrated in Figure 8b, according to the Mooney–Rivlin model, the presence of stress concentrations in areas covered by the thrombus is noticeable, specifically on the posterior side of the aneurysm.

In contrast, based on the HGO model, these stress concentrations are completely absent, which aligns with the previously mentioned literature regarding the contribution of the ILT to reducing the stresses of the aortic wall. The stress values are close to each other (up to 10% difference) for the unruptured and ruptured cases when applying the HGO model and increase with rising intraluminal pressure. For the unruptured case applying the Mooney–Rivlin model, both the stresses and strains are noticeably lower and never surpass the UTS limit. In the ruptured AAA geometry, the values are consistently higher than in the unruptured case, and the strength threshold (0.079 MPa) is exceeded for pressures higher than 192 mmHg.

## 4. Discussion

The current study introduces a detailed workflow to integrate a patient-specific, non-uniform ILT geometry into the biomechanical analysis of AAAs. Even though the ILT plays a pivotal role in AAA structural properties, many published computational studies overlook it. Clinically, the ILT’s structure does not always have distinct layers; its concentration can vary significantly within the aneurysmal sac [50]. Some regions might show minimal ILT, while others have a pronounced presence. The primary challenge for integrating the ILT into the AAA lies in the fact that the reconstruction and smoothing of the aortic wall and ILT lead to disconnected open surfaces that do not allow for computational meshing without further processing. Our approach, utilizing open software, ensures the seamless connection of the aortic wall and ILT surfaces, allowing the application of contact conditions at their common interface.

Another crucial point in the biomechanical analysis of AAAs (and more generally for aortic wall mechanics applications) refers to the implementation of the material model. The HGO model is considered one of the most representative models for such applications, but there is also an inherent difficulty in its implementation, as it mandates the designation of the collagen fiber orientation across the entire volume of the aorta/aneurysmal wall. To this end, a local Cartesian coordinate system for each element within the aortic wall mesh was applied, the implementation of which is not comprehensively presented in the literature. In addition to the reconstruction of the composite AAA geometry and the creation of a mesh-generation-ready 3D model, in the present work, information for the implementation of the HGO model is provided, with a special focus on the collagen fiber orientation.

Furthermore, this study delves into a dataset: medical images before and after the rupture of an AAA. At the initial assessment, the AAA’s diameter exceeded the surgical threshold by 30%. However, due to the patient’s advanced age and health profile, surgery was deemed too risky. Eventually, the AAA expanded and ruptured 20 months later. The results showed that both the unruptured and ruptured AAA models had similar maximum principal stresses, both exceeding the UTS in hypertensive states, as seen in Figure 9. However, the ruptured AAA consistently had a higher maximum principal stress across all pressure ranges. Notably, the maximum equivalent strain was also greater in the ruptured AAA, with this difference amplifying with increasing pressure. The highest principal stresses occurred where the thrombus was either minimal or non-existent. Similarly, peak equivalent strains appeared in areas devoid of ILT, showing an inverse relationship with ILT thickness. For the case under consideration, the rupture took place at a transitional area of the aortic wall, shifting from ILT-free to ILT-covered. Such transitional zones, beyond the rupture site, showed elevated stress levels. The observed stress concentration, meriting additional research, parallels findings by Xenos et al. [51].

**Figure 10:**
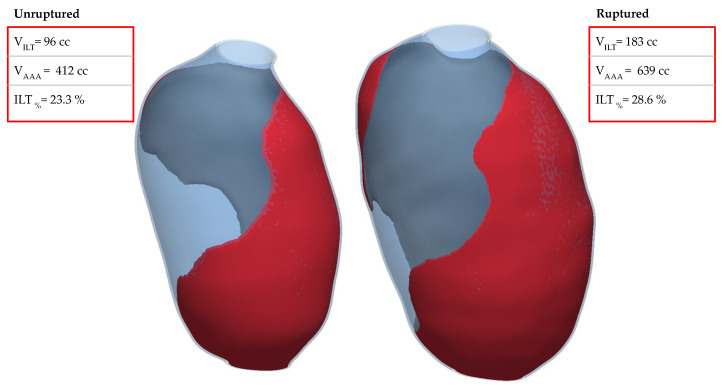
Volume parameters of AAA. (**left**) Unruptured, (**right**) ruptured.

Between the pre- and post-rupture assessments, there was an increase of the AAA volume from 412 mL to 639 mL and of the ILT volume from 96 mL to 183 mL, as depicted in Figure 10. Namely, the AAA and ILT volumes expanded by approximately 55% and 91%, respectively. However, the relative ILT volume ($ILT_{\%}$), namely, the ratio of the ILT volume to the aneurysm volume, increased from 23.3% to 28.6%. Several studies have underscored the significance of the AAA volume and relative ILT volume as robust predictors of the growth rate and rupture risk [52,53]. However, a comprehensive meta-analysis [54] posits that ruptured AAAs exhibiting larger ILT volumes in patients are likely attributable to a broader AAA diameter, rather than the ILT serving as a causative agent. This perspective supports the contested notion that the presence of an ILT is more incidental to the AAA than a primary contributory factor. Interestingly, in the current case, where the diameter exceeded the known thresholds, the $ILT_{\%}$ did not align with the range of 28.00–45.40% for asymptomatic and 37.70–54.40% for ruptured AAAs observed in a recent study of 98 patients [55]. Intriguingly, the $ILT_{\%}$ in the case at hand deviated from the range specified in the mentioned study, with values below the lower limits for the unruptured and ruptured cases. Given that the maximum principal stresses were also beneath the UTS in both states (unruptured and ruptured), this particular AAA case appears to diverge from conventional statistical patterns. Still, a rupture did occur, but it remains indeterminate whether the patient experienced acute stress, such as a hypertensive event, which would facilitate and explain this AAA complication. These observations underscore the inherent complexities in individualized rupture prediction.

## 5. Limitations

This study generally resonates with prior research [20,23] indicating that the thrombus’s presence may positively influence the mechanical loading of the aortic wall. However, factors such as aortic wall weakening from hypoxia and inflammation were not considered. It is important to highlight that integrating biochemical modeling necessitates a clearly defined and “mesh-able” ILT region where additional equations, like those for oxygen dissipation, can be defined and resolved. Model-wise, the study used an isotropic linear elastic model for the ILT, differing from others that utilized poroelastic [26,27] or isotropic hyperelastic models [33,56].

The aortic wall thickness was assumed to be constant, overlooking the variability in aortic thickness [42,43], due to the non-appearance of the wall contours on CT scans, not allowing its accurate measurement. Although this is a serious limitation, affecting the prediction of the wall stresses and strains, it is a standard practice due to lack of any other evidence.

The effects of aortic wall calcifications on the AAA stress concentration [57] were not taken into account. Their mechanical behavior in AAAs has not been fully clarified, despite their presence in 10% of adults above the age of 40 [58]. Nevertheless, the incorporation of calcifications is not a standard practice in AAA mechanical analyses. However, there are studies, such as Maier et al.’s work [57], where their influence is considered, and they are introduced into the geometry as well as into the wall material model following mechanical tests on samples of calcified aneurysmal aortic tissue.

The geometry obtained from the CT scans was assumed to be both unloaded and stress-free (following the conventional procedure), although this corresponds in the present work to a nonzero mean pressure of 100 mmHg. According to various published works that employed the so-called “zero pressure geometry”, the conventional procedure causes either an overestimation [59] or an underestimation of the predicted wall stresses [60]. As is shown in a recent numerical work [61], in addition to a zero-pressure geometry, it is important to exclude the residual stresses as well. However, the focus of the present work is mainly on the presentation of the details in the implementation of the HGO model and the treatment of the ILT–aortic wall interface.

FSI was not employed, considering the small amplitude of the AAA wall motion within the cardiac cycle which was measured to be 1.4% of the aneurysm diameter in a cohort of 56 patients [62], as a result of the reduction in the aorta compliance along its length [63,64] and the lack of elastin because of aging [64,65]. Collectively, these oversights represent the primary limitations of the present research.

## 6. Conclusions

The decision-making process in AAA treatment offers significant room for improvement. One of the many factors playing a crucial role in the progression of AAAs is the ILT. The inclusion of the ILT for the purpose of examining its overall impact has been the subject of research activity in recent years, with each investigation focusing on different aspects. The focal points of this work comprise a combination of the goals and approaches in relevant prior research, prioritizing the precision in the modeling of the ILT and the aortic wall. Our foremost contribution resides in elucidating a methodology, leveraging open software, to reconstruct patient-specific geometries, which may encompass non-uniform or even discontinuous thrombus geometries, to assess the mechanical impact of the thrombus on the AAA stresses and strains. Simulation outcomes suggest that the ILT acts akin to a reinforcing agent. This leads to reduced stresses in regions where its presence is most prominent, contrasting with ILT-free zones.

## Figures and Tables

**Table 1:** Material models’ parameters and values.

Body	Material Model	Parameter	Value	Dimensions
ILT	Linear elastic	*E*	0.11	MPa
		ν	0.45	(-)
Aortic wall	HGO	*c*	$300.55\times 10^{-3}$	MPa
		$k_{1}$	$16.32\times 10^{-3}$	MPa
		$k_{2}$	1768.15	(-)
		κ	0.1010	(-)
		α	5.6	(°)
	Mooney–Rivlin	$c_{1}$	$174\times 10^{-3}$	MPa
		$c_{2}$	$1881\times 10^{-3}$	MPa

The parameters of the HGO model, as averaged values of the aneurysmal wall for ruptured AAAs, were adopted from the work of Niestrawska et al. [49].

**Table 2:** Mesh sensitivity analysis parameters.

	Elements	Nodes	Max Stress Value (MPa)	Absolute Deviation at Max Stress
Coarse	247,948	64,939	0.0808	2.18%
Fine	427,252	107,054	0.0826	-
Finer	559,152	138,285	0.0837	1.42%

## Data Availability

Data are contained within the article.
